# Donut Morphology of Cardiac Amyloidosis: A Glazed Over Diagnosis

**DOI:** 10.1016/j.jaccas.2026.108679

**Published:** 2026-09-23

**Authors:** Pushan Aggarwal, Craig Alpert, Daniel Davies

**Affiliations:** a Medicine Institute, Allegheny General Hospital, Pittsburgh, Pennsylvania, USA; b Cardiovascular Institute, Allegheny General Hospital, Pittsburgh, Pennsylvania, USA

**Keywords:** cardiac magnetic resonance, chronic heart failure, imaging

## Abstract

**Background:**

Transthyretin cardiac amyloidosis (ATTR-CM) typically manifests with diffuse biventricular wall thickening, and technetium-99m pyrophosphate scintigraphy is highly accurate in this setting. False-negative results may occur, however, in early-stage disease when amyloid infiltration is nondiffuse.

**Case Summary:**

A 74-year-old man with heart failure with preserved ejection fraction, atrial fibrillation, and bilateral carpal tunnel syndrome was referred after negative cardiac amyloid radionuclide imaging (CARI) performed elsewhere. Cardiac magnetic resonance revealed isolated circumferential basal wall thickening with localized subendocardial late gadolinium enhancement and extracellular volume of 38%, forming a donut-shaped morphology. Repeat CARI with technetium-99m pyrophosphate confirmed nondiffuse grade 2 uptake in the basal left ventricle, establishing wild-type ATTR-CM. Tafamidis 61 mg daily was started alongside optimized heart failure therapy.

**Discussion:**

Amyloid deposition progresses base to apex, producing nondiffuse scintigraphy patterns in early disease. Cardiac magnetic resonance identified the unusual morphology and directed repeat scintigraphy, extending beyond current diagnostic guidelines and avoiding diagnostic delay.

**Take-Home Message:**

When clinical suspicion for ATTR-CM is high, negative CARI should prompt further evaluation rather than premature exclusion of the diagnosis.

## Visual Summary

**Figure:**
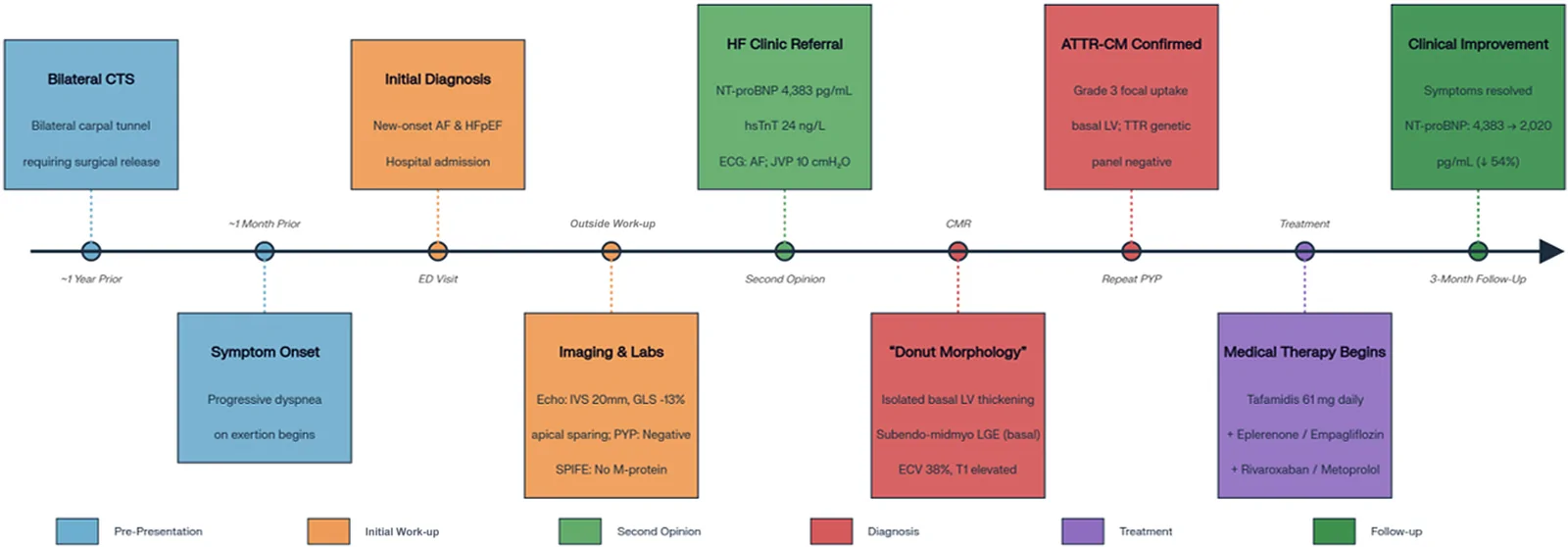

## History of Presentation

A 74-year-old man was evaluated in cardiomyopathy clinic for a second opinion. One month prior, he was diagnosed with new-onset atrial fibrillation and a first diagnosis of heart failure during an emergency department visit for progressive exertional dyspnea. He was seen in his local outpatient cardiology clinic and managed on loop diuretics and oral anticoagulation. Transthoracic echocardiography performed at an outside facility showed a borderline left ventricular ejection fraction (LVEF) of 50%, increased left ventricular (LV) wall thickness (up to 20 mm), and no significant valvular disease by report. The presence of apical sparing on LV global longitudinal strain prompted cardiac amyloid radionuclide imaging (CARI), which was reportedly negative. He remained symptomatic, which prompted referral for further evaluation.

**Figure 1:**
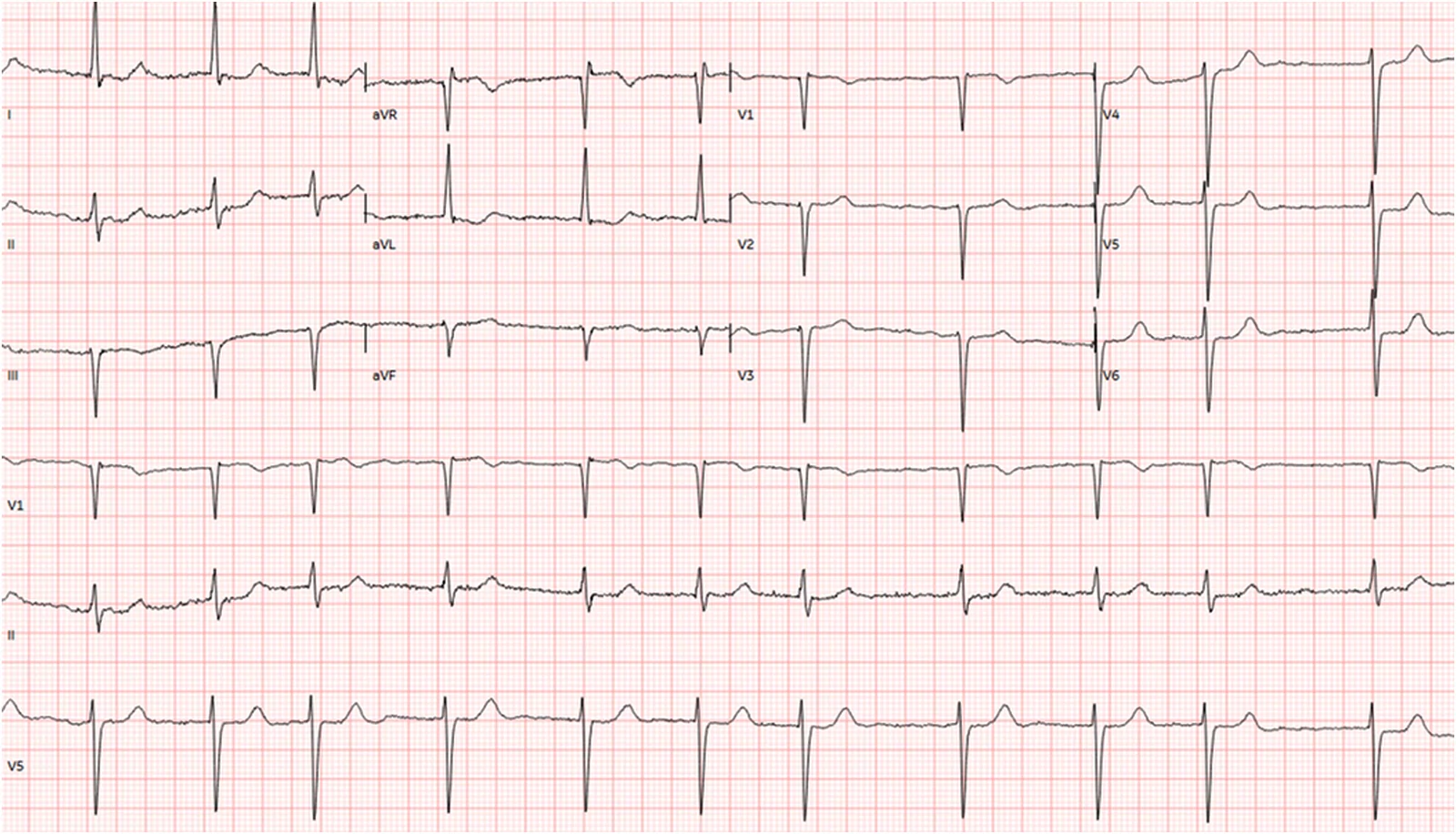
Electrocardiogram Electrocardiogram reveals atrial fibrillation, normal voltage, poor R wave progression (pseudoinfarct pattern), and nonspecific ST-segment changes.

On presentation to our clinic, physical examination revealed mild volume overload with a jugular venous pressure of 10 cm H_2_O and trace bilateral lower extremity edema; his lungs were clear bilaterally. Heart rhythm was irregularly irregular, consistent with atrial fibrillation. Laboratory data were notable for N-terminal pro–B-type natriuretic peptide 4,383 pg/mL, high-sensitivity troponin T 24 ng/L, hemoglobin 11.8 g/dL, and creatinine 1.08 mg/dL. Electrocardiogram showed an irregularly irregular rhythm, poor R-wave progression with a pseudo-infarct pattern, and non-specific ST-segment changes (Figure 1).

## Past Medical History

He reported a history of bilateral carpal tunnel syndrome requiring bilateral surgical release 1 year prior.^1^ He had no symptoms of peripheral neuropathy, spinal stenosis, gastrointestinal disease, or orthostatic hypotension. He denied a history of hypertension, diabetes, and chronic kidney disease or a family history of heart failure or cardiomyopathy.

## Differential Diagnosis

Taking into consideration the combination of LV wall thickening, a restrictive filling pattern, and carpal tunnel syndrome, the working differential diagnosis included transthyretin cardiac amyloidosis (ATTR-CM) (wild-type or hereditary disease) or amyloid light chain (AL) amyloidosis, hypertrophic cardiomyopathy (HCM), hypertensive cardiomyopathy, and Fabry disease. The exclusion of AL amyloidosis was prioritized given its distinct management implications. Nonobstructive HCM and Fabry cardiomyopathy were considered less likely owing to the absence of other morphologic cardiac features and family history, normal renal function, and the presence of apical sparing strain. The absence of any hypertension history argued against hypertensive cardiomyopathy.

## Investigations

Outside evaluation included a transthoracic echocardiogram with increased LV wall thickness (interventricular septum 20 mm, posterior wall 17 mm), borderline preserved LVEF of 50%, reduced right ventricular systolic function, biatrial enlargement, a trace pericardial effusion, and global longitudinal strain of −13% with an apical sparing pattern raising concern for cardiac amyloidosis.^2^ Pyrophosphate (PYP) scintigraphy at the referring institution was reported as negative, with no myocardial tracer uptake. However, the images and imaging protocol details (ie, use of single-photon emission computed tomography [SPECT] or SPECT/computed tomography [CT]) were not available for review. Serum and urine immunofixation electrophoresis were negative for a monoclonal protein with a normal kappa/lambda ratio, excluding plasma cell dyscrasia.^3^ Coronary angiography was negative for obstructive disease.

**Figure 2:**
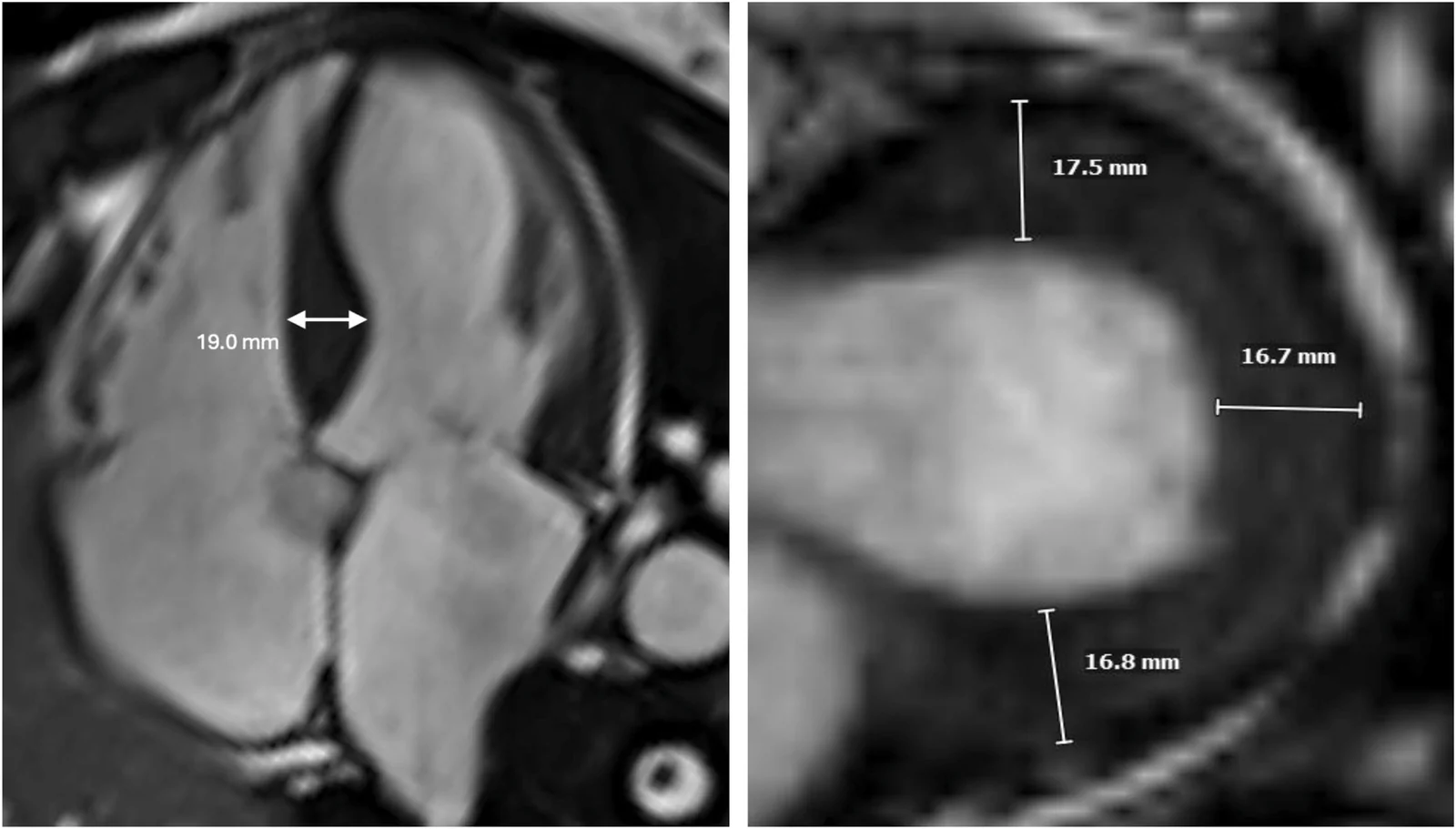
T1-Weighted Cine CMR Images T1-weighted cine (steady-state free precession) images from cardiac magnetic resonance (CMR) demonstrate isolated basal left ventricular wall thickening (19 mm basal septum, 17 mm basal inferolateral wall) with otherwise normal wall thickness in the mid–left ventricular to apical left ventricular walls, right ventricular free walls, and atrial walls.

**Figure 3:**
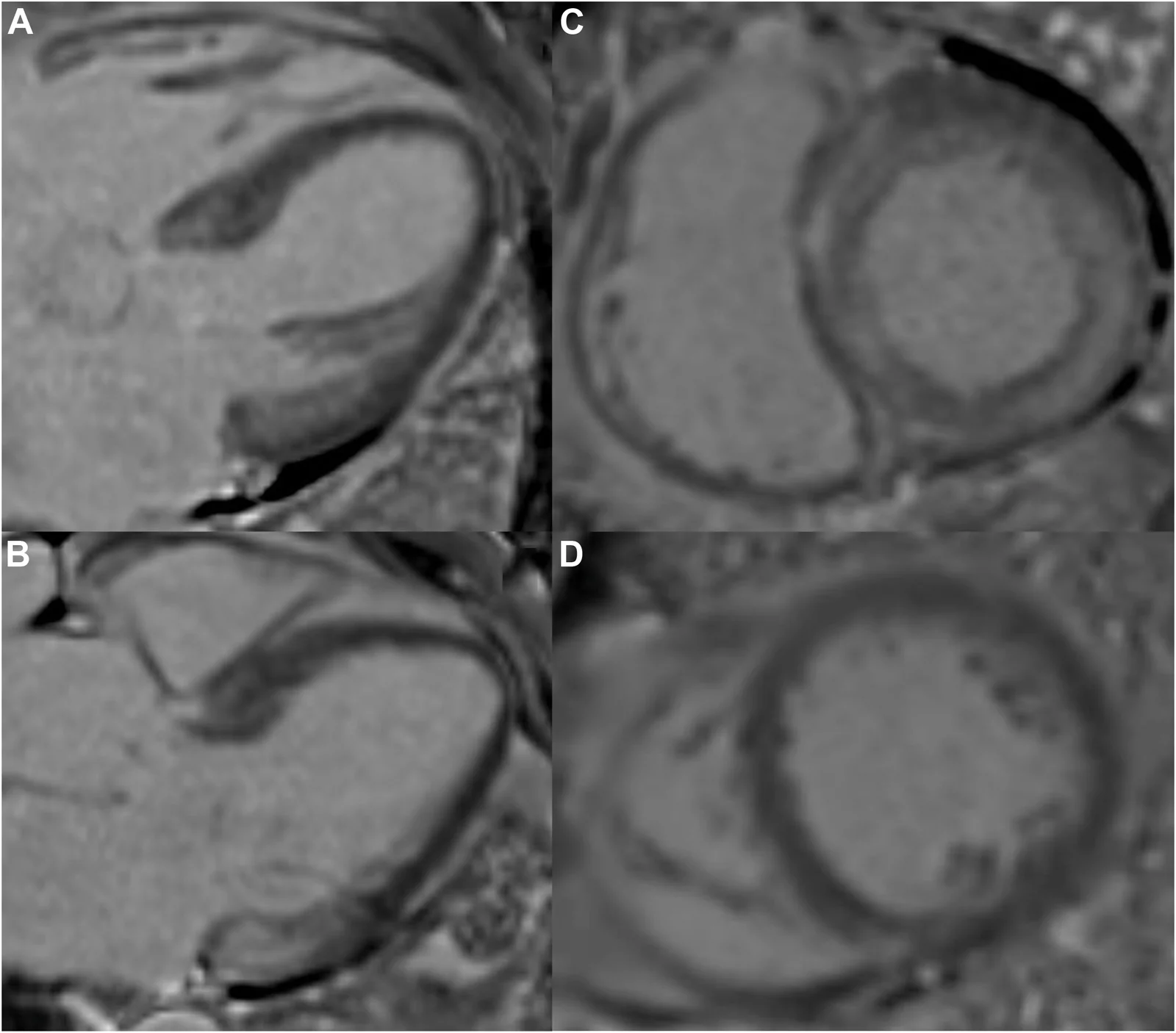
Delayed Post-Gadolinium Contrast CMR Images Delayed post-gadolinium contrast images from cardiac magnetic resonance (CMR) show prominent near-circumferential enhancement of the basal left ventricular segments (A to C) and basal right ventricular free wall (A and C). There is additional mild subepicardial enhancement at the mid-inferior right ventricular insertion site (D) with otherwise normal-appearing myocardium in mid-segments to apical segments. A trace simple pericardial effusion is visualized along the basal lateral heart (A to C).

**Figure 4:**
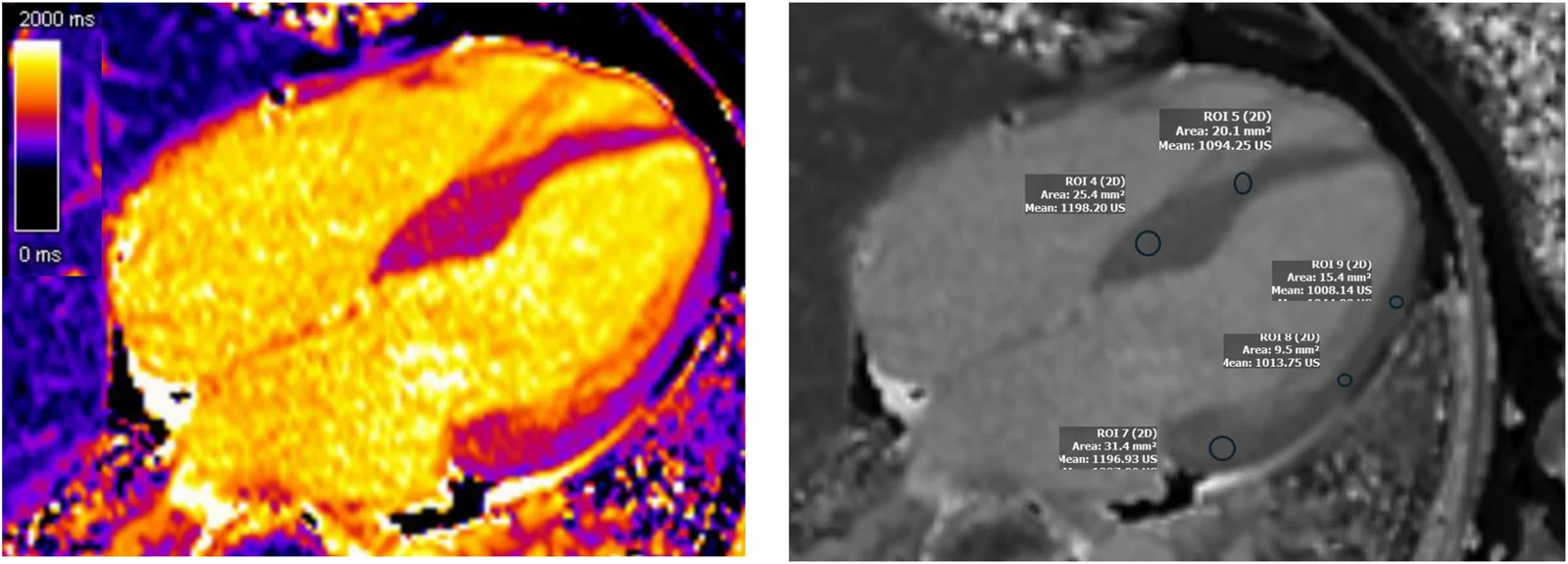
T1 Parametric Mapping From CMR T1 parametric mapping from cardiac magnetic resonance (CMR) shows prominent elevation of native T1 values in the basal segments (up to 1,198 ms) with a base-to-apex gradient of more normal values throughout the mid- and apical myocardium. ROI = region of interest.

Given persistent clinical suspicion for an infiltrative cardiomyopathy despite the negative outside PYP, cardiac magnetic resonance (CMR) was performed at our institution. This showed a mildly dilated LV (LV end-systolic volume index 117 mL/m^2^) with preserved biventricular systolic function (LVEF 54%, right ventricular ejection fraction 53%) (Videos 1A and 1B). Wall thickening was present, but only in an isolated circumferential pattern in the basal segments (Figure 2) measuring at most 19 mm in the basal septum and 17 mm in the basal inferolateral wall. Postcontrast imaging showed a normal global myocardial nulling pattern (Video 2) with prominent subendocardial to mid-myocardial late gadolinium enhancement (LGE) in the basal LV and right ventricular free walls, sparing the mid and apical myocardium (Figures 3A to 3D).^4^ Native T1 and extracellular volume (ECV) were mildly elevated in the affected basal segments, with a basal septal native T1 of 1,198 ms and ECV of 38%, consistent with early interstitial infiltration (Figure 4), again sparing the more apical heart.^4^ Taken together, this isolated basal pattern of wall thickening and late enhancement produced the distinctive donut-shaped morphology described here.

**Figure 5:**
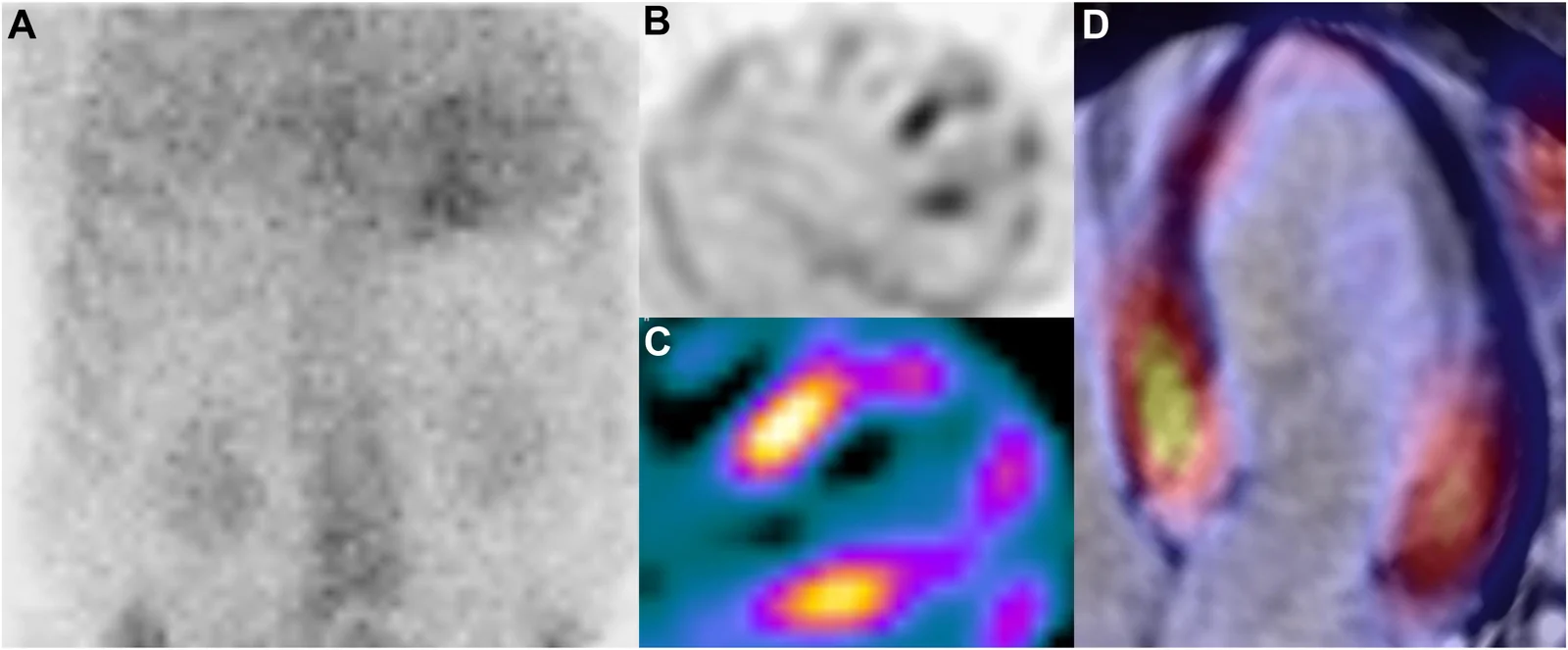
Technetium-99m Pyrophosphate Imaging in Cardiac Amyloidosis Cardiac amyloid radionuclide imaging performed at 1 hour with technetium-99m pyrophosphate shows a subtle abnormality on planar imaging (A) with activity adjacent to the sternum. On single-photon emission computed tomography images (B and C), nondiffuse (grade 2) myocardial uptake is clearly visualized in the basal left ventricle with more muted (grade 1) uptake in the mid-septum, closely matching the distribution of late enhancement on fused Cardiac amyloid radionuclide imaging/cardiac magnetic resonance late gadolinium enhancement images (D).

The CMR findings were thought to be most suggestive of early-stage cardiac amyloidosis or an atypical nonobstructive HCM phenotype with prominent fibrosis. Owing to concern of false-negative CARI, imaging was repeated with technetium-99m PYP at our institution instead of pursuing endomyocardial biopsy. On this examination, SPECT demonstrated grade 2 uptake isolated to the thickened basal LV with more subtle mid-ventricular uptake, closely mirroring the LGE distribution seen on CMR (Figures 5A to 5D).^3^^,^^5^ An extended cardiomyopathy genetic panel, including the *TTR* gene, was unremarkable without pathogenic variants, confirming without biopsy the diagnosis of wild-type ATTR-CM. At diagnosis, the patient was classified as National Amyloidosis Centre stage 2 and Grogan (Mayo) stage 3, reflecting an intermediate-to-advanced biomarker risk profile despite the early morphologic phenotype.

## Management

The patients was started on tafamidis 61 mg orally once daily for wild-type ATTR-CM,^6^ and heart failure therapy was further optimized with titration of loop diuretics and the addition of eplerenone and empagliflozin. Treatment with rivaroxaban and low-dose metoprolol succinate for atrial fibrillation was continued.

## Outcome and Follow-Up

At 3-month follow-up, the patient reported meaningful symptomatic improvement and return to his prior level of recreational activity. N-terminal pro–B-type natriuretic peptide had declined from 4,383 pg/mL to 2,020 pg/mL. He continued to experience persistent atrial fibrillation, but heart rate was well controlled, and he tolerated tafamidis and other therapies without adverse effects.

## Discussion

ATTR-CM is an increasingly recognized cause of heart failure with preserved ejection fraction, particularly in older men, and its prevalence in broader structural heart disease populations has increased substantially.^1^ The typical imaging findings include diffuse, concentric biventricular wall thickening and a characteristic apical sparing pattern on strain imaging.^2^ Nuclear scintigraphy with CARI radiotracers is highly accurate when uptake is diffuse, with near-perfect specificity when hematologic evaluation excludes AL amyloidosis.^3^^,^^5^ This case describes an atypical early morphologic variant that we term “donut morphology,” in which all imaging characteristics of amyloid heart disease were circumferentially isolated to the basal LV, a pattern not previously named to our knowledge in the literature.

This pattern may be more prone to produce false-negative imaging and may relate to the distribution of amyloid mass in the heart. Histopathologic studies have confirmed that total amyloid burden follows a base-to-apex gradient, with the greatest mass at the basal myocardium.^7^ Bravo et al,^7^ using ^18^F-florbetapir positron emission tomography and contrast-enhanced CMR in patients with cardiac amyloidosis, showed that although markers of total amyloid mass (extracellular LV mass and total florbetapir binding) demonstrated a marked base-to-apex gradient, the tissue concentration of amyloid measured by ECV and florbetapir retention showed no significant regional variation. These findings suggest that regional differences in strain and wall thickening are driven by the disproportionately greater total amyloid mass in the thicker basal segments and mid-segments, rather than by a higher proportion of amyloid infiltration at the base. In the context of this case, the mildly elevated ECV of 38% in the basal LV, combined with normal apical wall thickness, is consistent with early-stage disease in which total amyloid mass has not yet accumulated sufficiently to produce morphologic or functional abnormalities beyond the base.^7^

The report of negative CARI from an outside facility almost certainly reflects the challenges of localized myocardial uptake. When tracer uptake is limited to the basal segments, overall myocardial uptake on planar imaging falls below the visual grade 2 threshold, and heart/contralateral lung ratios may remain normal.^5^^,^^8^ Data from the hallmark study by Gillmore et al^2^ are instructive on this point: Among patients evaluated with CARI (PYP, 3,3-diphosphono-1,2-propanodicarboxylic acid, or hydroxymethylene diphosphonate), 1 of 8 with a grade 0 scan still had biopsy-confirmed ATTR-CM after excluding AL amyloidosis, and 10 of 11 patients with grade 1 scans had ATTR-CM. This suggests that absent or low-grade uptake on CARI does not reliably exclude disease when pretest probability is high.^3^ False-negative scans have similarly been reported in early-stage disease and in hereditary *TTR* variants, supporting the importance of routine SPECT or SPECT/CT with consideration of 3-hour imaging in all cases to minimize this risk.^8^

As the number of CARI-performing nuclear laboratories has expanded, several practical issues have contributed to an increase in inaccurate results in less experienced centers.^9^ Inaccuracy can occur with overreliance on planar-only imaging and quantitative planar assessment (heart/contralateral lung ratio) without close inspection of SPECT imaging. Planar imaging is limited by difficulty separating blood pool from myocardial uptake, bone uptake overlapping the cardiac silhouette, and variability in acquisition protocol and postprocessing region of interest application.^5^^,^^9^ The Perugini grading scale was designed for planar scintigraphy and evaluates uptake relative to bone in a diffuse pattern and may be more limited in its assessment of regional or nondiffuse myocardial uptake.^3^^,^^5^^,^^9^ Any myocardial PYP uptake on SPECT should be treated as abnormal and merits further evaluation, regardless of the visual grade on planar imaging.^5^ Routine use of SPECT or SPECT/CT for anatomical localization, standardized acquisition protocols, and broader recognition of nondiffuse uptake patterns are essential steps toward reducing diagnostic errors.^9^ When nondiffuse myocardial PYP uptake is identified, the differential diagnosis extends beyond early ATTR-CM to include AL amyloidosis, myocardial infarction scar, pericarditis, and chemotherapy-related myocardial injury, and appropriate follow-up with CMR and exclusion of a monoclonal protein process is essential.^5^

In this case, follow-up imaging with CMR drove further assessment toward the correct diagnosis. Whereas the pattern of basal-only thickening and myocardial changes was abnormal, the presence of circumferential (though localized) subendocardial LGE and elevated ECV led to the persistent suspicion of amyloidosis. CMR with LGE and T1/ECV parametric mapping provides important diagnostic and prognostic information in patients with suspected amyloidosis. Prior studies have shown that ECV outperforms longitudinal strain in distinguishing cardiac amyloidosis from other hypertrophic phenocopies, and T1 mapping adds prognostic data beyond what the LGE pattern provides alone.^4^ Martinez-Naharro et al^10^ demonstrated that ECV has high diagnostic accuracy for ATTR-CM (AUC: 0.91) and, importantly, that patients with equivocal grade 1 bone scintigraphy uptake had abnormal T1 and ECV values, suggesting that CMR can detect a phenotype of early amyloid infiltration even when scintigraphy is indeterminate. In our case, the characteristic morphologic CMR features prompted reconsideration of the initial nuclear result and ultimately avoided the need for endomyocardial biopsy.

The clinical picture also made it difficult to accept a negative scan at face value. Age over 70, male sex, bilateral carpal tunnel syndrome, atrial fibrillation, heart failure with preserved ejection fraction, and apical sparing on global longitudinal strain together represent multiple converging red flags that constitute high pretest probability for ATTR-CM under current consensus guidance.^1^^,^^5^ These features justified continued diagnostic pursuit despite the initial negative result. The donut morphology seen on CMR may represent an identifiable subset of early ATTR-CM in which the nondiffuse pattern itself should prompt repeat or confirmatory scintigraphy rather than acceptance of a negative scan.

## Conclusions

Isolated basal wall thickening with a donut-shaped LGE pattern on CMR represents an early, nondiffuse variant of ATTR-CM that is prone to producing false-negative PYP scintigraphy. When clinical pretest probability remains high despite a negative scan, CMR with LGE, T1 mapping, and ECV provides the spatial characterization needed to direct repeat nuclear imaging and avoid diagnostic delay.

**Visual Summary:**
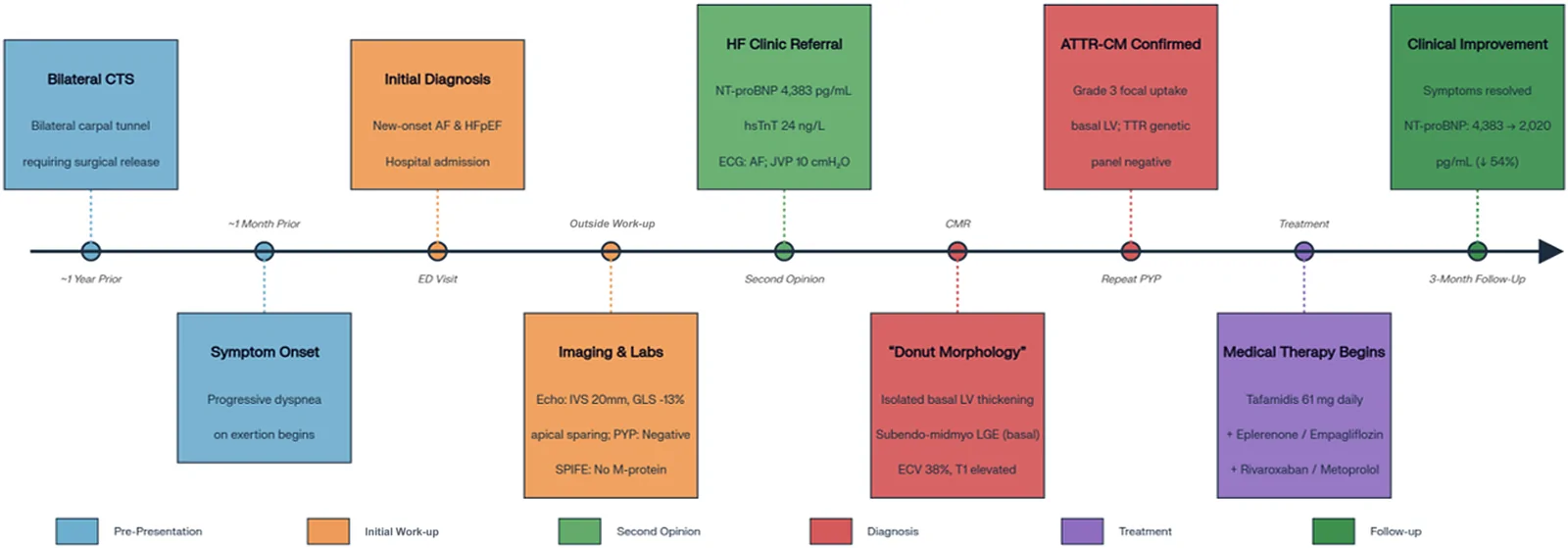
Timeline of Clinical Events Timeline of clinical events from initial presentation and outside evaluation, through CMR-based identification of donut morphology, repeat scintigraphy confirming the diagnosis, initiation of tafamidis, and clinical improvement at 3-month follow-up. AF = atrial fibrillation; ATTR-CM = transthyretin cardiac amyloidosis; CMR = cardiac magnetic resonance; CTS = carpal tunnel syndrome; ECG = electrocardiogram; ECV = extracellular volume; ED =emergency department; GLS = global longitudinal strain; HF = heart failure; HFpEF = heart failure with preserved ejection fraction; hsTnT = high-sensitivity cardiac troponin T; IVS = interventricular septum; JVP = jugular venous pressure; LGE = late gadolinium enhancement; LV = left ventricle/ventricular; NT-proBNP = N-terminal pro–B-type natriuretic peptide; PYP = pyrophosphate; SPIFE = serum protein immunofixation electrophoresis; subendo-midmyo = subendocardial to mid-myocardial.

## Funding Support and Author Disclosures

Dr Davies has consulted for Alnylam and BridgeBio. All other authors have reported that they have no relationships relevant to the contents of this paper to disclose.

**Take-Home Messages**

• A negative CARI scan does not exclude ATTR-CM in patients with high pretest probability; CMR with LGE and T1/ECV mapping should be considered when initial scintigraphy is negative or equivocal.
• Nondiffuse, basal-predominant amyloid infiltration on CMR (donut morphology) represents a recognizable early variant of ATTR-CM that should prompt repeat scintigraphy rather than premature exclusion of the diagnosis.

## Appendix

Video 1 T1-weighted cine images (steady-state free precession) from cardiac magnetic resonance imaging show prominent, isolated wall thickening in the basal myocardium. (A) 3-chamber view. (B) 4-chamber view. There was normal biventricular systolic function and the absence of resting left ventricular outflow tract flow acceleration and systolic anterior motion of the mitral valve (A).

Video 2 T1-weighted cine (steady-state free precession) images from cardiac magnetic resonance imaging demonstrate isolated basal left ventricular wall thickening (19 mm basal septum, 17 mm basal inferolateral wall) with otherwise normal thickness of the mid to apical left ventricle, right ventricular free walls, and atrial walls.
